# Health Tract, No. 305

**Published:** 1867-09

**Authors:** 


HEALTH TRACT, No. 305.

### REST.

Multitudes of earth's toiling millions have died while striving to make enough money to retire from business, and in a beautiful cottage on their own little farm to spend the remnant of their days in rest, in having nothing in particular to do. Perhaps one in a million of the hopers does make money enough to enable him to retire to his country-seat, and for a year or two, while he is fixing it up to his notion, all goes on charmingly, but when everything is completed to his mind and he has nothing more to take up his attention, he eats and sleeps and lounges around for a few months longer, falls into disease and dies; or if he has unusual force of character and power of observation, he notices that both health and happiness are passing from him and tracing this to the true cause of an inactive body and an unoccupied mind, he resolves to "sell out" and plunge again into the vortex of business.

Recently an old school-mate,—younger, graduating in the same class thirty-seven years ago—writes that "both body and mind are worn out; the slightest physical labor exhausts him," and, "any effort to think or study or even read, so wearies the brain that life is felt as a burden." He withdrew from his professional duties, which he had performed in the place for twenty-five years, with honor to himself, having secured the love and confidence and respect of all who knew him. He gave up his calling for the purpose of obtaining rest, as a means of health.

The number of families is increasing every day, who give up housekeeping as a means of rest from family cares, and resort to that miserable and most unwise mode of life, boarding at a hotel or in some private family, to get more dissatisfied than ever in a few months, meanwhile falling into bad health and bad habits of various kinds.

All these classes of persons fail, miserably fail in their object because they mistake the physiological meaning of the word "rest." Neither body nor brain are safely, truly and happily rested by doing nothing. The only healthful rest, as long as our physical and mental constitution remains as it is, is to be busy. Men of force and industry will everywhere tell you, "It is the hardest thing in the world to do nothing." No mortal man was ever made to be a loafer, to be a miserable drone. The true idea of rest is recreation, a making over again, a return to our accustomed vigor; and this is accomplished, not by allowing the machine to come to a stand-still, for inactivity is rust and ruin to all machanical contrivances, and death to all physiological structures. The true object of rest is recuperation, and that is best brought about as to the body, by exercising a different set of muscles; and as to the brain by calling into requisition a different set of organs or powers, causing the mind to act upon new objects. A better plan is not to get into the unhealthful conditions named, and they are avoidable by giving two hours daily to the exercise of a different class of muscles or to the investigation and study of objects of comparatively trivial importance and of a wholly different nature. The student should ride on horseback, or cultivate fruits and flowers; the merchant should employ his mind in liberal studies, in active personal and elevating charities, while the over-taxed and worried wife should pay a visit daily to some prudent friend, some cheery neighbor or suffering sister or child;—the main idea in all cases being to spend two or three hours daily in open-air activities wholly different from the ordinary business routine.—From the Boston Watchman and Reflector.

